# Aortic valve fenestrations: a review

**DOI:** 10.1097/j.pbj.0000000000000083

**Published:** 2020-09-16

**Authors:** Caixia Zhu, Sofia C. Torres, José Pedro L. Nunes

**Affiliations:** a Faculdade de Medicina da Universidade do Porto; b Centro Hospitalar Universitário São João, Porto, Portugal.

**Keywords:** aortic insufficiency, aortic regurgitation, aortic valve fenestrations, review

## Abstract

**Background:**

Aortic valve fenestrations (AVFs) seem to be relatively common; however, their impact in human heart disease is not entirely clear.

**Methods:**

A review was carried out to assess all scientific literature on human patients related to AVFs, as described in the published literature. The search was conducted on 2 different databases, Medline (PubMed), and ISI Web of Knowledge.

**Results:**

Fifty-five reports were under analysis. Autopsy studies showed AVFs to be present in 55.9% of individuals studied in such studies. They occur more frequently in men and, in general, their frequency increases with age. Although common, fenestrations rarely cause regurgitation; however, they may play an important role in the pathophysiology of some cases of severe aortic regurgitation. AVFs have been described in patients with Down syndrome and Marfan syndrome, in patients with bicuspid or quadricuspid valves, and in patients with myxomatous valvular degeneration. Echocardiographic assessment of aortic regurgitation seems to have limitations in the diagnosis of valvular fenestrations.

**Conclusions:**

Fenestrations of the aortic valve are very common and are associated with certain clinical conditions. It is unknown if AVFs play any role in the current epidemic of aortic valve disease. Future studies should aim to better define the role of AVFs in aortic valve disease, to further understand its etiology and to develop diagnostic criteria.

## Introduction

Aortic valve (AV) disease is increasingly prevalent and has a varied etiology. AV fenestrations (AVFs) have been recognized as a cause of AV disease for more than a century.^1–3^ Although they are not a rare finding, AVFs are currently seen as a relatively infrequent cause of valve disease, namely of AV regurgitation (AR).

Early data^4^ points in the direction that AVFs are seen in more than half of examined hearts. The most frequent site of the fenestrations is adjacent to the attachment of the free edge of the cusp to the aortic intima. Fenestrations are usually ovoid apertures, with the long axis parallel to the free edge of the valve. Their frequency increases with age up to the fourth decade of life, they are more common in men and multiple fenestrations could be seen in the same patient.^4^

Fenestrations have been seen in AVs that exhibit features of myxomatous degeneration^5–14^; however, congenital fenestrations can also occur.^4,6,7,15–20^

We conducted a review to assess all scientific literature on human patients related to AVFs, as described in the published literature.

## Methods

We conducted a review according to the guidelines for Preferred Reporting Items for Systematic Reviews and Meta-Analyses.

### Search strategy

A comprehensive review of the literature was performed to identify all reported articles on AVFs. The search was conducted on 2 different databases, Medline (PubMed) and ISI Web of Knowledge, in July 2019. The search queries in both databases were as follows: “aortic valve fenestration”; “aortic valve fenestrations”. No restrictions concerning date of publication were imposed. The lists of references of studies included in the final analysis were also manually searched.

### Inclusion criteria

The review considered all human studies reporting on AVFs. Both prospective and retrospective human studies were included.

### Exclusion criteria

We excluded articles that were not available in English or Spanish, as well as reports regarding animals (nonhuman) and publications with no original data.

### Study eligibility assessment

Study eligibility was individually assessed by 2 investigators. No formal quality assessment was carried out, since a significant number of articles were case reports.

**Figure 1:**
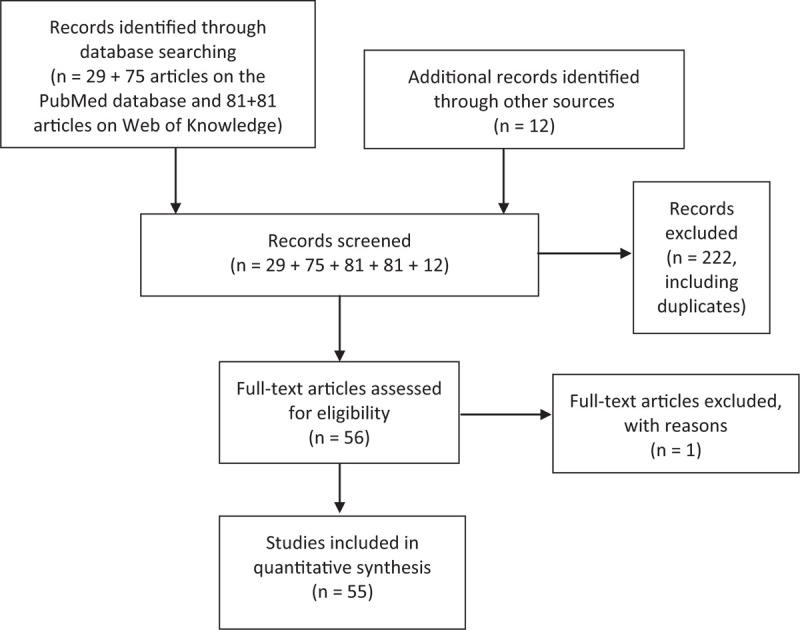
Flowchart showing literature search method. n = number of articles.

Full text of 7 articles were unavailable, one^21^ of which was not included in Table 1           for that reason, while the remaining six^22–27^ were considered, since they appeared to contain relevant information. The data of four^24–27^ was obtained from the respective abstracts and two^22,23^ from other cited reports (Fig. 1).^8,28^

**Table 1:**
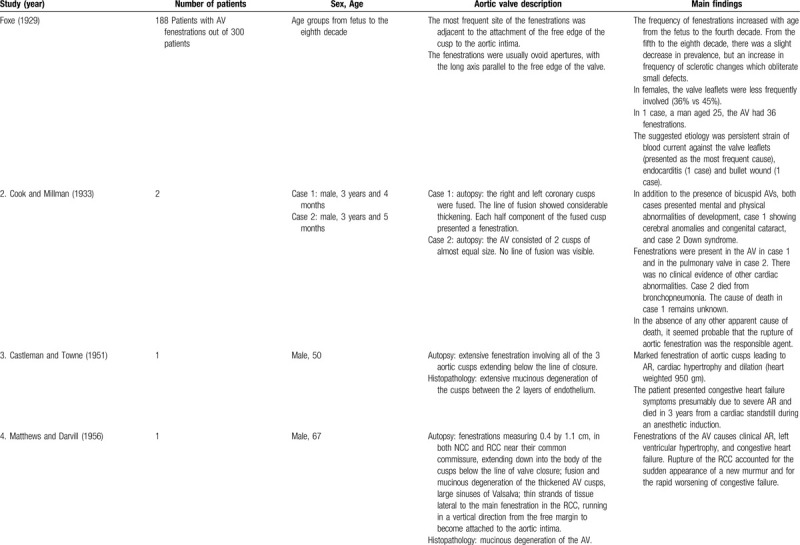
List of published reports on aortic valve fenestrations

**Table 1:**
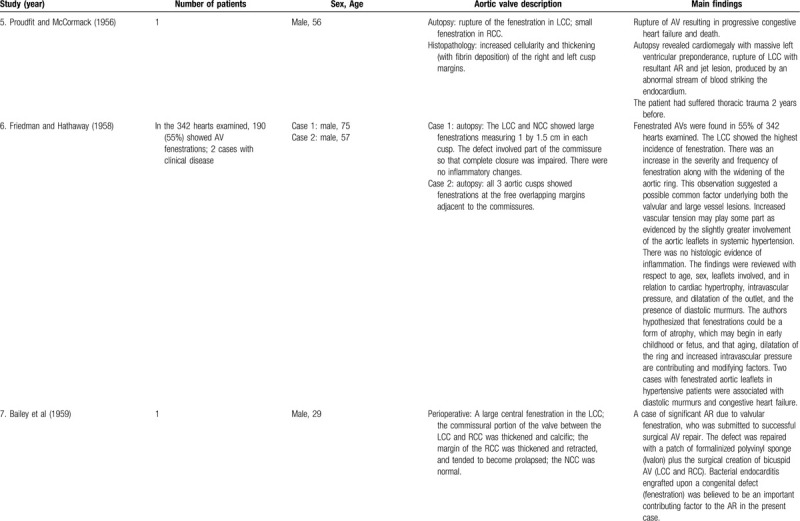
List of published reports on aortic valve fenestrations

**Table 1:**
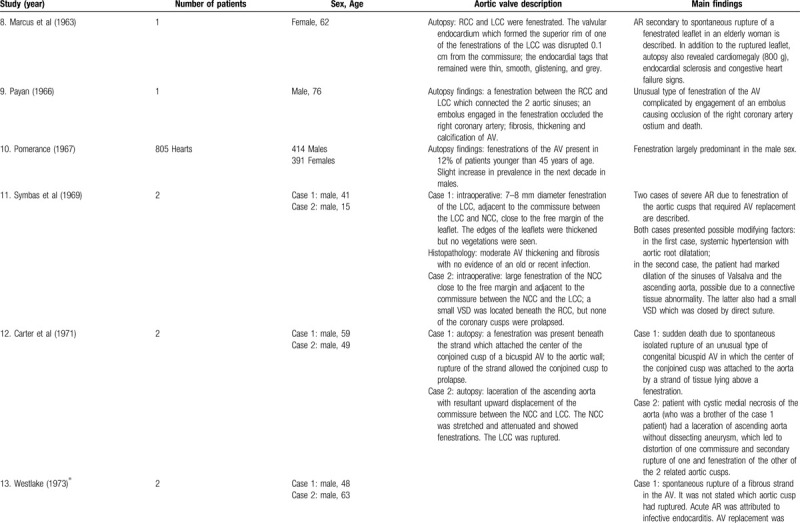
List of published reports on aortic valve fenestrations

**Table 1:**
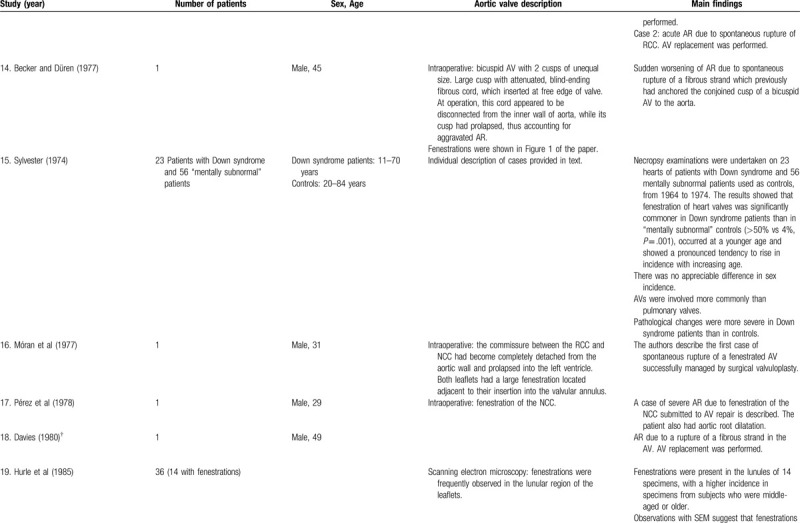
List of published reports on aortic valve fenestrations

**Table 1:**
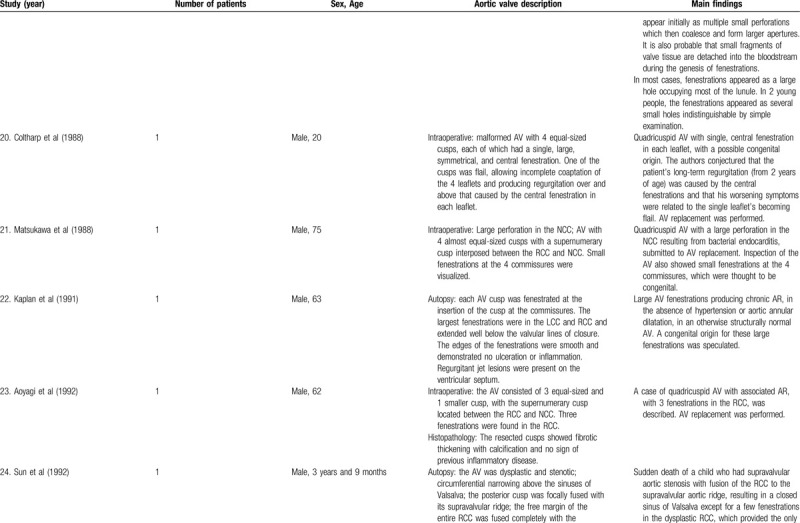
List of published reports on aortic valve fenestrations

**Table 1:**
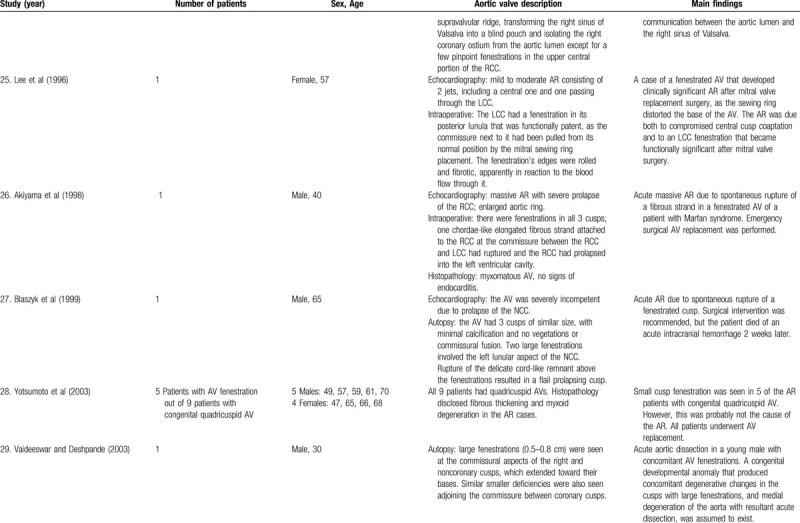
List of published reports on aortic valve fenestrations

**Table 1:**
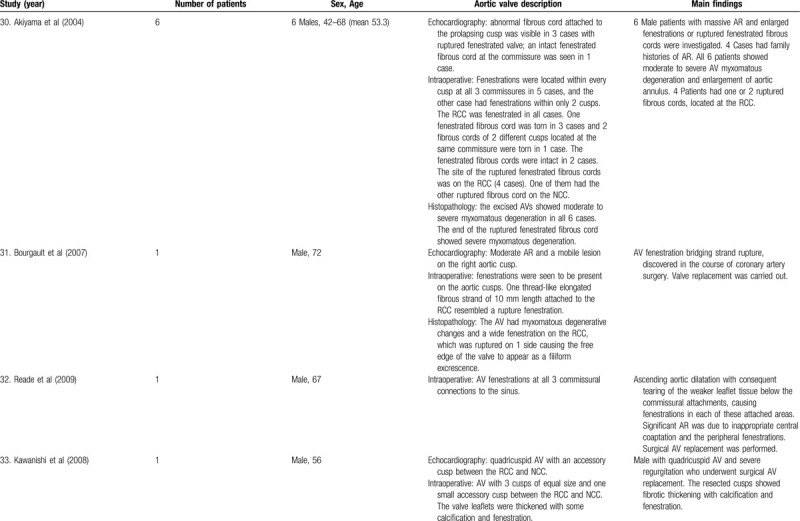
List of published reports on aortic valve fenestrations

**Table 1:**
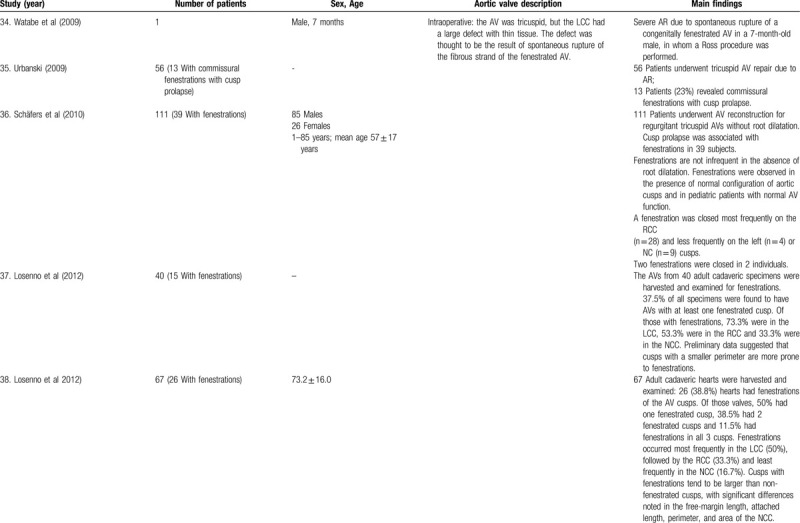
List of published reports on aortic valve fenestrations

**Table 1:**
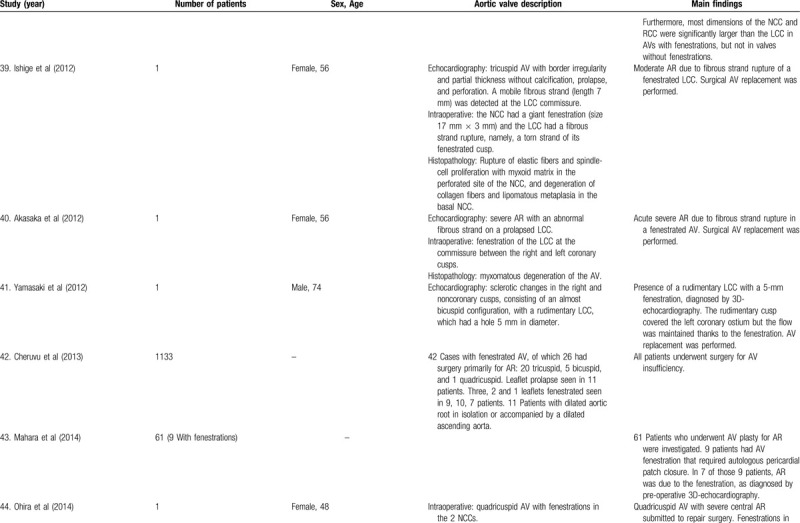
List of published reports on aortic valve fenestrations

**Table 1:**
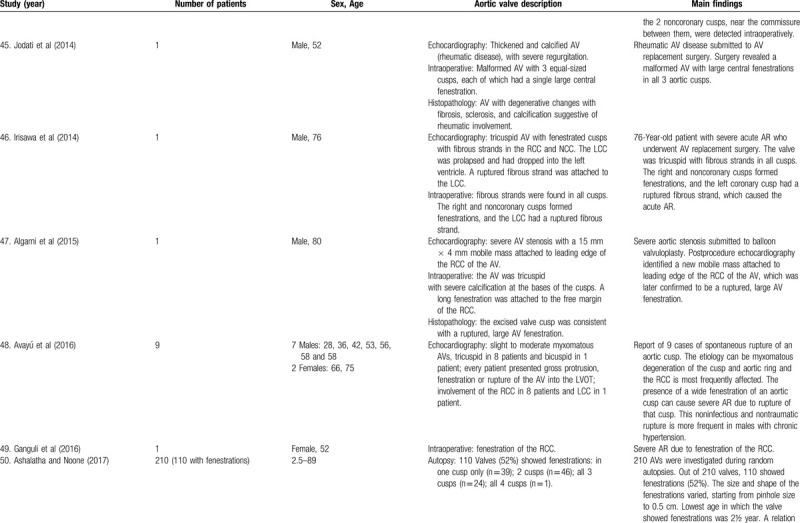
List of published reports on aortic valve fenestrations

**Table 1:**
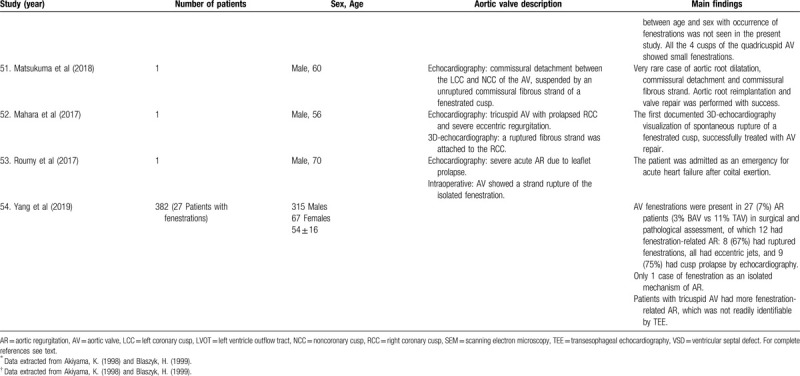
List of published reports on aortic valve fenestrations

### Study selection and data extraction

The investigators individually assessed if studies addressed the topic under study, and if all inclusion/exclusion criteria prespecified for the review (as described above) were met. This was done initially through title and abstract analysis, and then (if abstracts complied to specifications) through full-text assessment. Any discrepancies were resolved by consensus between the authors. Data extraction was performed independently by 2 authors using a predesigned extraction form. The following items were extracted from each study by 2 authors (C.Z. and S.T.) independently and presented in the Table 1          : author, year, number of patients, sex, age, AV description, and main findings.

## Results

**Figure 2:**
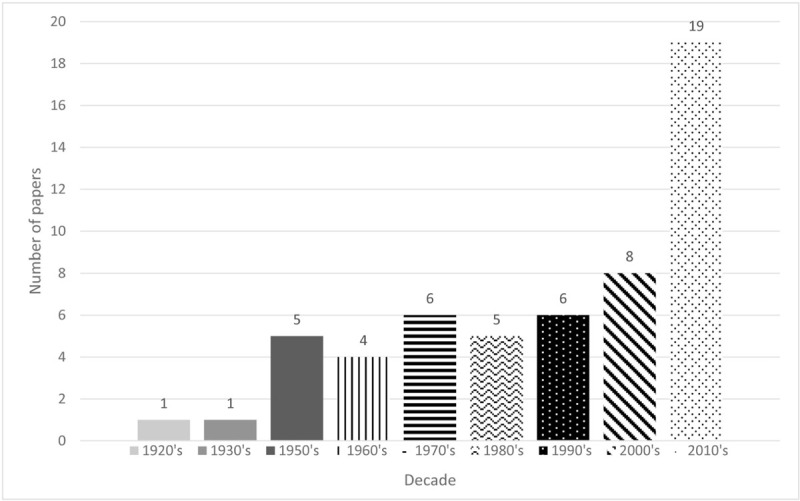
Classification of research papers by year of publication.

We selected 29 articles with the first query and 75 articles with the second query on the PubMed database, and 81 articles with the first query and another 81 articles with the second query on ISI Web of Knowledge. Twelve additional records were identified through other sources. Of the 278 records screened, 222 were excluded after title and abstract review as not meeting the inclusion criteria. A total of 55 articles were included in our study, according to the selection criteria. In Table 1           we present the data for 54 of these articles, for which we were able to obtain data. As shown in Figure 2, only 1 study was published in the 1920s,^4^ 1 from the decade of 1930,^29^ 5 from the decade of 1950,^5–7,20,30^ 4 from the decade of 1960,^31–34^ 6 from the decade of 1970,^22,35–39^ 5 from the decade of 1980,^19,21,23,40,41^ 6 from the decade of 1990,^8,9,18,28,42–44^ 8 from the decade of 2000,^10,11,16,17,45–48^ and 19 from the decade of 2010.^12–15,24–27,49–59^

With regard to study design, 39 (70.9%) were case reports, 3 (5.5%) were small case series, 7 (12.7%) studies were cross-sectional, 5 (9.1%) were cohort studies (including 1 prospective cohort study and 4 retrospective cohort studies), while 1 (1.8%) study was a cross-sectional study which included also case reports.

### Prevalence of aortic valve fenestrations in autopsy studies

We identified 4 larger series of patients, corresponding to autopsy studies.^4,7,24,57^ The prevalence of fenestrations in AV in these 4 reports is summarized in Table 2. Out of a total number of 919 examined hearts, 545 hearts exhibited AVFs. Therefore, we estimate that the prevalence of fenestrations in AVs is 55.9%.

**Table 2:**
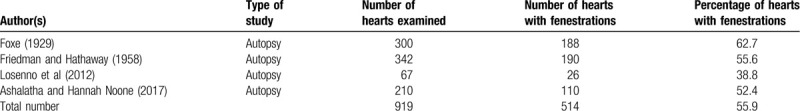
Prevalence of fenestrations in aortic valves in 4 autopsy studies.

Foxe^4^ described a series of 188 (62.7%) cases of AVFs, out of 300 cases studied. According to this author, the most frequent site of the fenestrations was adjacent to the attachment of the free edge of the cusp to the aortic intima, and the fenestrations were usually ovoid apertures, with the long axis parallel to the free edge of the valve.^4^ Further major findings are presented in Table 1          . Friedman and Hathaway^7^ examined 342 hearts, of which 72% had semilunar valve fenestrations; 2 cases had clinical disease. Fenestrations in the AV were seen in 190 cases (55.6% of the total number of hearts).

Losenno et al^24^ studied 67 cadaveric human hearts, of which 26 (38.8%) had fenestrations in the AV. The results showed that fenestrations occurred most frequently in the left coronary cusp (LCC) (50%), followed by the right coronary cusp (RCC) (33.3%) and least frequently in the noncoronary cusp (NCC) (16.7%). The authors studied the dimensions of the AV cusps with and without fenestrations and concluded that cusps with fenestrations are generally larger than nonfenestrated cusps. In addition, the NCC and RCC tend to be larger than the LCC in AVs with fenestrations, but not in valves without fenestrations. The authors conjectured that the fenestrations could develop as a result of unequal shear stress on the cusps of eccentric AVs.

Ashalatha and Hannah Noone^57^ reported on AVs from 210 random autopsy cases. Tricuspid AVs (TAVs) existed in 208 cases. Fenestrations were seen in 110 valves (52.4%). The authors described variations in size and shape of the fenestrations. There were 2 cusps affected in 22% of cases, and 1 cusp in 19% of cases. When 1 cusp alone showed fenestrations, the LCC was the most commonly affected and NCC was the least affected.

Pomerance^33^ described a series of 805 hearts studied at autopsy. Fenestrations of the AV were present in 12% of patients younger than 45 years, with an increase in prevalence in the next decade in males. Precise figures on the overall prevalence of fenestrations were not given by the author.

### Case reports and small case series

Of the 39 case reports and 3 small case series, there were a total of 63 patients with AVFs. Four (6.3%) patients had bicuspid AVs (BAVs), 45 (71.4%) patients had TAVs, 10 (15.9%) patients had quadricuspid AVs (QAVs) and in 4 (6.3%) patients the number of AV cusps was not specified. The RCC had at least 1 fenestration in at the minimum 38 patients, the LCC was affected in at least 23 patients, the NCC was affected in at least 24 patients, the conjoined in BAVs was affected in 2 patients, and the supernumerary cusp in QAVs was affected in at least 4 patients. Taking into consideration the 45 patients with TAVs, 11 (24.4%) had fenestrations in all cusps. Concerning the 10 patients with QAVs, at least 4 (40%) had fenestrations in all cusps.

At the minimum, 28 patients had ruptured fenestrations in the AV, 21 were reported to have arterial hypertension (of which 19 had chronic arterial hypertension), 18 had no arterial hypertension, and in 24 this topic was not specified.

### Fenestration-related aortic regurgitation or stenosis

AVFs were seen in a large research study of patients with AR conducted by Yang et al.^59^ The authors noted that of the 382 patients undergoing surgery for moderately severe and severe aortic insufficiency, 12 (3.1%) had fenestration-related AR (2 with BAV and 10 with TAV), but only 1 (0.26%) exhibited AVFs as an isolated mechanism of AR. Out of these 12 cases, 8 (86.7%) had ruptured fenestrations, 9 (75.0%) had cusp prolapse and all had eccentric jets. The authors also recognized that fenestrations with/without rupture as an AR etiology in TAV were underdiagnosed by transesophageal echocardiography because of its limitations in detection of small perforations and free-edge/paracommissural fenestrations.

Cheruvu et al^51^ described a series of 1133 patients who had AV surgery, of which 42 had valve fenestrations, and 26 of those had surgery primarily for AR.

One case of AV stenosis^54^ and 1 case of supravalvular aortic stenosis were described in association with valve fenestrations^9^ (Table 1          ).

### Aortic valve fenestrations in Down and Marfan syndromes

AVF was seen in association with Down syndrome. Sylvester^36^ conducted an investigation to study the incidence of semilunar valve fenestrations in 23 patients with Down syndrome and in 56 “mentally subnormal” subjects, used as controls. Sylvester identified 10 patients with Down syndrome (43.5%) who exhibited AVFs, whereas in the control group only 2 patients (3.6%) had these features in the valvular leaflets (not specified in which semilunar valve). In addition, the author described that there was a significant increase in frequency with increasing age. A case of AVF was described in association to Marfan syndrome.^8^

### Fenestration in bicuspid and quadricuspid aortic valves

As already mentioned above, fenestrations were also seen in association with BAVs. We found 4 case reports^14,29,35,37^ and 2 cases in the above mentioned study of Yang et al,^59^ in which fenestrations were present in BAVs. All the 6 patients had fenestration-related AR, of which 3^14,35,37^ had spontaneous rupture of the fenestrated AV.

Cases of fenestrations in QAVs have also been identified, with at least 10 case reports^19,41,42,46,53^ and 1 small case series^10^ in the literature. All the cases described patients with AR, but unlike the cases of BAVs, only 1 case^41^ had its underlying cause attributed to valvular fenestrations, while in the remaining 9, AR was due to other mechanisms.

### Myxomatous degeneration

Many published articles have described myxomatous degeneration in AVs with fenestrations. There are at least 9 case reports and series^5,6,8–14^ that found myxomatous changes in the affected cusps. Friedman and Hathaway^7^ described in their autopsy study that several aortic leaflets showed myxomatous degeneration in varying degrees; however, it was not possible to make a definite correlation between any of the changes seen in microscopic study and the presence of fenestrations.

## Discussion

In the present report, we undertook a review concerning AVFs. These fenestrations are commonly located in the commissures, they are considered a frequent condition and their existence is well established for more than a century^1,2^; however, their clinical relevance and possible complications have not been clarified yet.

In our review, we assessed the published articles on this subject, and our results show that an estimate of 55.9% of individuals has fenestrations in aortic leaflets, according to autopsy studies (Table 2). The incidence of fenestrations is considerably higher in men than in women.^7^ Its frequency increases with age up to the fourth decade^4,7^ and slightly decreases from the fifth decade on, which can be explained by the development of sclerotic lesions that obliterate small defects.^4^

Many authors speculate that the changes caused by myxomatous degeneration in the AV are responsible for the development of the fenestrations; nevertheless, this correlation has not been firmly demonstrated. For instance, Friedman and Hathaway observed similar degenerative alterations in healthy valves without fenestrations. Therefore, the presence of myxomatous degeneration per se is not sufficient to cause these effects, and the contribution of other factors must be considered. In 3^4,7,24^ of the 4 biggest patient series, the authors hypothesized that, due to the eccentric anatomic configuration of some AVs,^24^ the turbulent shear stress distribution of the blood flow on the leaflets leads to the development of the fenestrations. Moreover, it has been hypothesized that chronic arterial hypertension in some patients might play some role in the etiology of this type of valvular injury, by interacting with the dynamics of the blood stream and creating a tensile stress on the cusps. Nonetheless, it does not explain the existence of fenestrations in valves that are not under such stress, hence chronic hypertension does not seem to be an essential etiologic factor.

On the contrary, Foxe visualized fenestrations in fetal hearts, along with other authors who reported several cases of congenital etiology.^6,7,15–20^ Thus, defects during embryonic development may contribute to the adult pattern now described.

Other etiologies such as ulcerative endocarditis,^4,19,20,22^ and traumatic origin^4,30^ were also identified. However, there is no consensus among the authors as to whether these defects due to secondary causes should be considered as fenestrations. Such a perspective would point to a mechanical etiology for some cases of fenestrations.^4^

In addition, many articles have recognized fenestrations as the space limited by a fibrous strand which anchors the free edge of the cusps to the inner wall of the aorta. In this context, the opening formed in atypical bicuspid AV where a fenestrated raphe joins the valve cusp to the aortic wall is also included in the classification of fenestration.

Currently, the concept of valvular fenestration is not well defined in the literature and is often used as a synonym of perforation, hole, or as an atypical form of bicuspid AV with a fenestrated raphe. Standardized classification criteria for valvular fenestrations in medical terminology are needed to clarify the distinction between this condition and other similar features in future articles.

Although fenestration is a common feature, it rarely causes clinical manifestations or has influence on AV competence. Among the causes of aortic insufficiency, fenestrations account for only 3.1% of AR mechanisms.^59^ Nonetheless, regurgitation can occur in cases of large valvular fenestrations,^7,11,15,17,18,20,28,34,38,41,54^ particularly in large central ones,^15,20,41^ and in spontaneous rupture of fenestrated AVs, causing sudden appearance or worsening of a chronic AR.^6,8,11–14,16,22,23,27–31,35,37,38,45,52,54,56,59^ Iatrogenic rupture of a fenestration caused by balloon aortic valvuloplasty was also identified.^54^ It has been suggested that the presence of associated modifying factors, such as aortic root dilatation, can trigger fenestration-related AR. Aortic root dilatation, which reduces the height of the subcommissural triangles and the coaptation area of the cusps, leads, as a consequence, to the incorporation of the fenestration into the functional portion of the valve. Incompetence through the fenestration may hence develop, allowing the subsequent presence of a regurgitation jet.^7,31^

Sylvester pointed at an association between Down syndrome and the number of fenestrations in the AV. These were significantly commoner in patients with Down syndrome (43.5%) than in patients with normal karyotype (3.6%), occurred at a younger age and showed a pronounced tendency to rise in incidence with increasing age. The most obvious limitation in this research was the small sample size, which prevented a clear generalized statement about this correlation.

Fenestrations were also seen in association with congenital bicuspid and quadricuspid AVs. Nevertheless, as there are no epidemiological data available concerning the present subject matter and the number of cases reported in the literature is limited, it was not possible to reach conclusive results. Further studies are necessary to assess the incidence of fenestrations in nontricuspid AVs and its possible complications, namely AR.

Concerning diagnostic tests, although AVFs are common features, they are not promptly diagnosed by echocardiography. Because of their anatomic location (coaptation zone or paracommissure), visualization of fenestrations by transesophageal echocardiogram is relatively difficult, meaning that a negative echocardiogram alone cannot exclude their presence. Thus, fenestration-related AR is generally identified during surgical inspection. However, ruptured fenestrations frequently show leaflet prolapse, which could be a clue to their presence.^59^ Another suggestive echocardiogram finding is the visualization of a mobile fibrous strand attached to an aortic cusp near its commissure, particularly in a prolapsing leaflet. Although fenestrations rarely cause valvular incompetence, they should be considered in the differential diagnosis of AR, particularly in male patients with chronic AR or acute deterioration of the regurgitation, in the absence of any other plausible cause.

It is unknown whether AVFs play any role in the current epidemic of AV disease, namely if the presence of 1 or more fenestrations changes the incidence of clinical AV disease, either stenosis or regurgitation. Currently, this topic seems to be largely overlooked, not being mentioned in current guidelines.^60^ Future research may perhaps establish if the incidence of AV stenosis is changed in association to the previous presence of valve fenestrations.

### Limitations

Texts written on non-English or Spanish languages were not considered. We were unable to obtain 7 full texts from the selected reports, one of which^21^ was not included in Table 1           for that reason. Four reports were included in Table 1           with the data based on the respective abstracts.^24–27^ Concerning the other 2 reports,^22,23^ the presented data were obtained from other cited reports.^8,28^ Marked differences were seen in the reports under analysis.

## Conclusions

Autopsy studies showed AVFs to be relatively common, being present in 55.9% of individuals studied in such studies. They occur more frequently in men and, in general, their frequency increases with age. Fenestrations rarely cause marked regurgitation; however, they may play an important role in the pathophysiology of some cases of severe aortic insufficiency. AVFs have been described in patients with Down syndrome, in patients with bicuspid or with quadricuspid valves, and in patients with myxomatous valvular degeneration.

It is unknown whether AVFs play any role in the current epidemic of AV disease. Future studies should aim to better define the role of AVFs in AV disease, to further understand its etiology and to develop diagnostic criteria.

## Acknowledgments

None.

## Conflicts of interest

The authors declare no competing interests.
